# Comparative validation of speckle contrast optical spectroscopy against diffuse correlation spectroscopy for monitoring human cerebral blood flow

**DOI:** 10.1117/1.NPh.13.2.025008

**Published:** 2026-04-16

**Authors:** Tom Y. Cheng, Mitchell B. Robinson, Marco Renna, Kuan-Cheng Wu, Zachary Starkweather, Olivia S. Kierul, Byungchan (Kenny) Kim, Alexander C. Howard, David A. Boas, Stefan A. Carp, Xiaojun Cheng, Maria Angela Franceschini

**Affiliations:** a Massachusetts General Hospital, Athinoula A. Martinos Center for Biomedical Imaging, Department of Radiology, Boston, Massachusetts, United States; b Boston University, Department of Biomedical Engineering, Boston, Massachusetts, United States; c Massachusetts Institute of Technology, Lincoln Laboratory, Lexington, Massachusetts, United States

**Keywords:** speckle contrast optical spectroscopy, diffuse correlation spectroscopy, cerebral blood flow, two-layer phantom, pulsatile waveform

## Abstract

**Significance:**

Within diffuse optics, speckle contrast optical spectroscopy (SCOS) has emerged as a promising alternative to the state-of-the-art technique of diffuse correlation spectroscopy (DCS) for continuous, noninvasive bedside monitoring of cerebral blood flow (CBF). Although the two methods theoretically yield equivalent relative indices of CBF (rCBFi), in practice, SCOS-based measurements require experimental calibration to obtain unbiased rCBFi values. To date, there are limited validation studies comparing SCOS and DCS in human subjects, particularly at long source–detector separations (SDS) relevant to adult brain monitoring.

**Aim:**

We aim to compare rCBFi from SCOS and DCS during concurrent, colocalized measurements on tissue-mimicking phantoms and humans at a large SDS of 3 cm.

**Approach:**

We conducted concurrent SCOS and DCS measurements on temperature-ramped, two-layer, and flow phantoms, respectively. We also conducted concurrent CBF measurements on 10 healthy volunteers undergoing various physiological challenges (breath-holding, hyperventilation, pressure modulation, and squatting) designed to elicit measurable changes in blood flow. SCOS and DCS operated from a shared pulsed laser source, which enabled pulsation-resolved human CBF measurements at 3 cm SDS (DCS necessitated the use of cardiac-gated averaging).

**Results:**

We observed strong agreement (r=0.93, slope = 0.98) between SCOS- and DCS-derived rCBFi at 3 cm SDS and an order-of-magnitude improvement in SCOS noise performance relative to DCS during the *in vivo* measurements. Phantom experiments showed a small depth-sensitivity disadvantage for SCOS at the same SDS; however, this was far outweighed by its superior noise performance, yielding an order-of-magnitude improvement in SCOS contrast-to-noise ratio.

**Conclusions:**

The results demonstrate the equivalence of properly calibrated SCOS and DCS rCBFi measurements at long SDS in adults, establishing SCOS as a viable alternative to DCS for monitoring CBF. Further validation across larger cohorts and clinical populations is warranted.

## 1 Introduction

Continuous monitoring of brain perfusion plays an important role in guiding the treatment of evolving pathologies and reducing patient mortality in the neurocritical care unit.^1^ The gold standard for continuous brain perfusion monitoring is monitoring cerebral perfusion pressure—a surrogate for cerebral blood flow (CBF)—using invasive intracranial pressure and mean arterial pressure monitors.^2^ Noninvasive methods for CBF monitoring are attractive due to their lower risk of complications^3^ and availability outside neurosurgical centers, but current noninvasive methods used in the clinic, including computed tomography (CT), magnetic resonance imaging (MRI), and transcranial doppler (TCD) ultrasound, provide only temporal snapshots of CBF that limit clinicians’ ability to respond to changing cerebral physiology.^4^

Near-infrared diffuse optical approaches for measuring CBF are promising candidates for clinical translation, as they enable continuous, noninvasive bedside monitoring and help address the limitations of current noninvasive methods.^5^ Diffuse correlation spectroscopy (DCS), which measures tissue blood flow through analysis of the temporal intensity fluctuation of speckles formed by coherent, diffusely backscattered light,^6^ has been extensively validated *in vivo* against gold-standard techniques for measuring CBF^7^ and is increasingly used in clinical research.^8^ However, the clinical translation of traditional DCS has been limited by its low light throughput and suboptimal signal-to-noise ratio (SNR), particularly in adult populations.^9^ Recent advances in DCS technology—including interferometric detection,^10^^–^^12^ the use of longer wavelengths (i.e., 1064 nm),^13^ highly parallelized speckle detection,^14^^–^^17^ and time-of-flight discrimination^18^^–^^22^—have significantly improved SNR and cerebral sensitivity, although these enhancements come at the cost of increased instrumentation complexity.^23^

A related, more recently developed diffuse optical technique, speckle contrast optical spectroscopy (SCOS), has emerged as a promising tool for CBF monitoring.^24^^,^^25^ Similar to DCS, SCOS derives its blood flow signal from the flow-induced temporal dynamics of speckles. However, SCOS quantifies the rate of speckle evolution differently—by measuring the reduction in spatial speckle contrast across a speckle image over a given camera integration time, similar to the approach used in laser speckle contrast imaging (LSCI).^26^^–^^28^ Using readily available complementary metal-oxide-semiconductor (CMOS) cameras with millions of pixels, SCOS can detect several orders of magnitude more speckles in parallel than DCS, resulting in a dramatic increase in light throughput and SNR.^29^ In addition, thanks to tremendous advances made in the miniaturization and cost reduction of megapixel CMOS cameras, SCOS technology is accessible and has a relatively clear path toward affordable, wearable formats. This has led to a recent proliferation of increasingly performant, compact, and higher-channel-count SCOS devices for monitoring human CBF.^30^^–^^33^

Despite SCOS’s performance potential, the ability of SCOS to reliably provide CBF measurements equivalent to those of DCS remains understudied. Although the theoretical equivalence of SCOS and DCS has been established,^24^^,^^34^ in practice several sources of error in the SCOS signal must be controlled or corrected to obtain accurate blood flow measurements.^35^ Unlike DCS, which is typically calibration-free,^9^ SCOS requires experimental calibration to account for detection noise and camera nonidealities that bias its blood flow measurements.^36^

Several pioneering validation studies on SCOS have used tissue-mimicking phantoms,^29^^,^^37^^–^^43^ animal models,^40^^,^^44^^–^^46^ and human arm cuff occlusions.^25^^,^^37^^,^^39^^,^^40^^,^^42^^,^^43^^,^^47^^,^^48^ Encouraging results from these studies have laid important groundwork and supported the adoption of SCOS for human CBF monitoring. However, these findings may not directly apply to human CBF measurements due to differences in source–detector separation (SDS), tissue structure, physiology, and optical properties. To date, only two studies have validated SCOS CBF measurements against DCS in humans,^48^^,^^49^ using a 2 cm SDS, which generally provides low, if any, sensitivity to cerebral signals in adults.^50^ A recent study reported a good correlation between SCOS and TCD measurements (R=0.79, slope = 0.87) at a long SDS of 3.6 cm, enabled by using a short-pulse, high-peak-power laser.^31^ Although this result is encouraging, the study was limited to a single physiological maneuver (breath-holding), and there are concerns about using TCD as a reference for tissue-level CBF, as it measures blood flow velocity in the middle cerebral artery—a large, upstream vessel that does not directly reflect microvascular or cortical perfusion.^51^ In addition to the limited number of SCOS validation studies for human CBF, there is also a lack of experimental comparisons of depth sensitivity between SCOS and DCS.

To help address this gap, we performed concurrent, colocalized SCOS and DCS measurements at 3 cm SDS on phantoms and healthy volunteers undergoing physiological challenges (breath-holding, hyperventilation, pressure modulation, and squatting) designed to elicit large changes in measured blood flow. With proper camera calibration and bias correction, SCOS demonstrated excellent agreement with conventional DCS and showed over an order of magnitude improvement in noise performance *in vivo*.

## 2 Materials and Methods

### 2.1 Optical Instrumentation

**Fig. 1:**
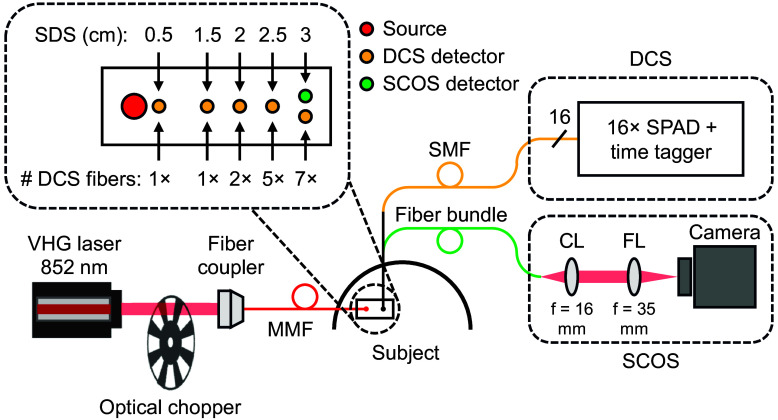
High-level diagram of the hybrid SCOS-DCS optical instrumentation used in this work. The probe depicted was used for human measurements. SDS: source–detector separation; DCS: diffuse correlation spectroscopy; SCOS: speckle contrast optical spectroscopy; VHG: volume holographic grating; MMF: multimode fiber; SMF: single-mode fiber; SPAD: single-photon avalanche diode; CL: collimating lens; FL: focusing lens.

The following sections describe the major components of the hybrid SCOS-DCS optical instrumentation shown in Fig. 1.

#### 2.1.1 Pulsed laser system

Our laser source was a continuous-wave (CW) 852 nm volume holographic grating (VHG) stabilized laser diode (Thorlabs LD852-SEV600). As first demonstrated in Ref. 30, we reduced the duty cycle of the laser to 10% using an optical chopper (Thorlabs MC2000B with MC1F2P10 blade, Newton, New Jersey, United States) after collimation of the CW laser diode, allowing us to increase the laser’s instantaneous power by 10 times relative to CW illumination. This increased the flow-induced speckle contrast and the SCOS measurement SNR by 10-fold.^36^ This strategy enabled us to measure pulsation-resolved human CBF at 3 cm SDS, using a chopping frequency of 25 Hz. A frequency of 25 Hz was also used for the two-layer phantom experiment, whereas a frequency of 20 Hz was used for the temperature-ramped phantom and flow phantom experiments.

We adhered to the ANSI Z136.1 laser safety standard for continuous skin illumination, using an average power of $P_{\mathrm{avg}}=36\;\mathrm{mW}$ ($P_{\text{peak}}=360\;\mathrm{mW}$) over a Ø3.5  mm aperture.^52^^,^^53^ The laser was shared between SCOS and DCS to enable direct comparison between the two techniques.

#### 2.1.2 Optical probe

The rectangular probe used for human subject measurements was 3D-printed using a black, flexible thermoplastic polyurethane (TPU) filament (NinjaTek NinjaFlex, Midnight Black, Pennsylvania, United States). The probe held the source fiber and both the DCS and SCOS detector fibers parallel to the tissue surface, and mirrors or prisms were used to redirect the light. As shown in Fig. 1, the probe was placed on the right side of the forehead, below the hairline, and ∼5  cm from the midline.

The source fiber was a Ø400  μm core multimode fiber (Thorlabs FT400EMT, Newton, New Jersey, United States). To reduce the optical power density and ensure skin and eye safety, we placed a holographic diffuser (Edmund Optics #55-852, Barrington, New Jersey, United States) at the fiber output. A Ø5  mm silver mirror (Edmund Optics #89-463) was used to deflect the source light out of the probe. We caution users that it is their responsibility to choose optical components, including fibers, mirrors, and diffusers that ensure their entire optical setup complies with applicable laser safety and regulatory guidelines.

For DCS detection, we used single-mode fibers (Thorlabs 780HP) positioned at SDSs of 0.5, 1.5, 2, 2.5, and 3 cm. To improve the DCS SNR, we bundled multiple fibers together at the longer SDSs, allowing for autocorrelation functions from several detectors to be averaged: 1× at 0.5 cm, 1× at 1.5 cm, 2× at 2 cm, 5× at 2.5 cm, and 7× at 3 cm. The phantom experiments used different numbers of DCS fibers per SDS as shown later. Silver-coated 1.5-mm right-angle prism mirrors (OPCO Laboratory, Inc., Massachusetts, United States) were used to direct light from tissue into the DCS collection fibers. Although the 0.5 cm SDS was intended to measure scalp blood flow, the 4-ms illumination pulse width was too short for the DCS autocorrelation function at this separation to fully decay. As a result, valid DCS measurements could not be obtained at 0.5 cm SDS. We retained this channel to facilitate detection of laser-on periods in the DCS data, as described later.

The SCOS detector fiber was a custom fiber bundle (0.66 NA, composed of ∼3700  Ø38  μm core multimode fibers, Fiberoptics Technology) with a 90 deg bent circular end (Ø2.5  mm) for tissue contact on the probe side and a rectangular end (1.64  mm×3  mm) for coupling to the SCOS camera. The SCOS fiber was positioned at 3 cm SDS, as close as possible to the DCS fiber at the same SDS (center-to-center spacing of 4 mm) to enable nearly colocalized measurements.

The probes used for phantom experiments were 3D-printed using a black, rigid polyethylene terephthalate glycol (PETG) filament (IC3D #RM-PE0011) and had holes to hold the ferrule-terminated source and SCOS/DCS detector fibers. To better approximate the semi-infinite geometry of human measurements, each fiber hole was surrounded by at least 1.5 cm of black material in all directions, preventing photon paths that travel above the measurement plane. The locations of the SCOS and DCS fibers on the phantom experiment probes are shown later.

#### 2.1.3 Diffuse correlation spectroscopy detection and data analysis

For DCS detection, we used a traditional homodyne DCS system with fiber-coupled silicon single-photon avalanche diode (SPAD) detectors (Excelitas SPCM-AQ4C, Pittsburgh, Pennsylvania, United States) and a custom field programmable gate array (FPGA)-based time tagger with a resolution of 6.7 ns.^54^ A total of 16 SPAD detectors were used and distributed across the different SDSs as shown in Fig. 1.

Photon detection timestamps corresponding to each 4-ms illumination pulse were identified by detecting the rising and falling edges of the pulses in the timestamp data. The edge detection was facilitated using the 0.5 cm SDS channel, which had the highest photon count rate, and by binning the data using a 100-μs bin width. Because all DCS detectors shared a common time base via the FPGA time tagger, the pulse timings identified from the 0.5 cm SDS channel were applied to the other DCS channels.

We used the photon timestamps from each pulse to compute an intensity autocorrelation function (ACF), using unbiased normalization to correct for the decreasing number of available autocorrelation values at longer time lags,

$$\mathrm{ACF}(m)=\frac{1}{N-|m|}\sum_{n=0}^{N-m-1}I(n)I(n+m),$$ (1)

where I is the discrete-time photon detection sequence of length N, and m is the time lag. The time lags were then logarithmically binned to reduce the noise in the ACF at longer time lags.^55^ We normalized the ACFs by the mean intensity squared ${\left\langle I\right\rangle }^{2}$ to obtain the normalized intensity autocorrelation function, $g_{2}(\tau )$.

The analytical solution to the correlation diffusion equation for a semi-infinite homogeneous medium was then fit to the $g_{2}(\tau )$ with known or assumed optical parameters (i.e., absorption and reduced scattering coefficients, refractive index, wavelength, and SDS) to estimate αD—the product of the probability of scattering from a moving scatterer (α) and the apparent diffusion coefficient (D)—which is regarded as the blood flow index (BFi) in DCS.^56^ For phantom experiments, $D=D_{B}$, the Brownian diffusion coefficient, and α≈1.

To ensure consistent fitting of the most information-rich range of the measured $g_{2}(\tau )$ across all evaluated flow rates and SDSs, we defined a dynamic fitting range based on the coherence parameter β. Specifically, we defined the fitting range as the τ interval over which $g_{2}(\tau )-1$ (after temporal averaging to 0.1 Hz) was between 95% and 5% of the β value. We chose these limits to avoid SPAD afterpulsing artifacts^57^ and excessive measurement noise at early time lags, as well as to avoid overly weighting the fit with the portion of the $g_{2}(\tau )$ at long time lags, which is less sensitive to the brain.^58^ To estimate β and the τ values corresponding to 95% and 5% of β, we fit a single exponential decay function $f(\tau )=\beta \;\exp(-\tau /\tau_{c})$ to $g_{2}(\tau )-1$. We then fit the previously mentioned correlation diffusion model to the $g_{2}(\tau )$ over the τ interval determined above to extract BFi (and a reestimate for β). Figure S1 in the Supplementary Material shows an example fit of the $g_{2}(\tau )$ using our method.

For the phantom experiments, we used values of the absorption coefficient ($\mu_{a}$) and reduced scattering coefficient ($\mu_{s}^{\prime }$) measured using frequency-domain near-infrared spectroscopy (FD-NIRS) instrumentation. For the human subject experiments, we assumed $\mu_{a}=0.16\;{\mathrm{cm}}^{-1}$ and $\mu_{s}^{\prime }=9\;{\mathrm{cm}}^{-1}$ at our wavelength of 852 nm.^13^^,^^59^ We used a refractive index value of n=1.33 (water) for all phantom experiments except for the two-layer phantom, where we used n=1.4 due to the addition of glycerol, which has a refractive index of n=1.47. For the human subject experiments, we assumed n=1.4.^7^^,^^60^^,^^61^

#### 2.1.4 Speckle contrast optical spectroscopy detection and data analysis

To perform SCOS measurements, we magnified and imaged the rectangular end of the SCOS detector fiber bundle onto a Basler a2A1920-160umPRO CMOS camera using an aspheric condenser collimating lens (f=16  mm, Thorlabs ACL25416U-B) and a plano-convex spherical focusing lens (f=35  mm, Thorlabs LA1027-B). To control and stabilize the camera’s temperature, we installed the manufacturer’s heat sink (Basler #2200000998) and operated the camera for ∼15  min before each measurement.^36^ The CMOS camera was previously validated for SCOS and characterized according to the procedure described in Ref. 36 to obtain accurate values for camera gain and pixel-wise dark noise, required for the SCOS noise correction process. The noise correction procedure used in this work largely follows the method described in prior work^30^^,^^36^ and is summarized below.

The photogenerated intensity value I was calculated for each pixel from the raw intensity value $I_{\text{raw}}$ and the dark intensity value $I_{\text{dark}}$,

$$I=I_{\text{raw}}-I_{\text{dark}}.$$ (2)

A region of interest (ROI) was defined within the image of the fiber for all speckle images. The ROI defined for the rectangular fiber bundle contained ∼1.77 million pixels, out of 2.35 million (1936×1216)  pixels available on the camera. Nonoverlapping 7×7  pixel windows were identified within the ROI, and windows with temporal mean intensity below a threshold of 25 DN were regarded as invalid and discarded. We implemented the intensity threshold to avoid low intensity levels associated with high nonlinearity in the camera’s photon transfer curve (PTC)—defined as the variance of the intensity $\sigma^{2}(I)$ versus the mean intensity ⟨I⟩—which can lead to large errors in the estimated shot-noise-induced speckle contrast, $K_{s}$.^36^

The raw speckle contrast $K_{\text{raw}}=\sigma (I)/\langle I\rangle$, where σ(I) is the spatial standard deviation of pixel intensities and ⟨I⟩ is the spatial mean of pixel intensities, was calculated for each valid 7×7  pixel window within the ROI. The flow-induced speckle contrast $K_{f}$ for each window was extracted from the raw speckle contrast $K_{\text{raw}}$ after calculating the sources of bias in the speckle contrast due to shot noise $K_{s}$, read noise $K_{r}$, spatially nonuniform illumination $K_{\mathrm{sp}}$, and quantization noise $K_{q}$:

$$K_{f}^{2}=K_{\text{raw}}^{2}-K_{s}^{2}-K_{r}^{2}-K_{\mathrm{sp}}^{2}-K_{q}^{2}.$$ (3)

The bias terms in Eq. (3) are given by,

$$K_{s}^{2}=g/\langle I\rangle ,$$ (4)

$$K_{r}^{2}=\sigma_{r}^{2}/{\left\langle I\right\rangle }^{2},$$ (5)

$$K_{\mathrm{sp}}^{2}=\sigma_{\mathrm{sp}}^{2}/{\left\langle I\right\rangle }^{2},$$ (6)

$$K_{q}^{2}=1/12/{\left\langle I\right\rangle }^{2},$$ (7)

where g is the camera gain, $\sigma_{r}$ is the camera read noise, and $\sigma_{\mathrm{sp}}^{2}$ is the spatial variance of the temporal mean image within the window. Strictly speaking, our camera read noise term $\sigma_{r}$ included the dark shot noise because we estimated $\sigma_{r}$ from a series of dark images.^36^ For the camera exposure time used in this study (6 ms, longer than the laser pulse width), the dark shot noise was negligible compared with the read noise.^35^^,^^59^

For improved estimation accuracy of $K_{s}^{2}$, especially at lower intensity levels or if errors due to camera PTC nonlinearity are nonnegligible, $K_{s}^{2}$ can be estimated directly from a high-resolution measurement of the camera’s PTC. A procedure for PTC characterization was previously described in Ref. 36. Assuming incoherent and uniform illumination ($K_{f}^{2}=0$ and $K_{\mathrm{sp}}^{2}=0$), $K_{s}^{2}$ is given by,

$$K_{s}^{2}=K_{\text{raw}}^{2}-K_{r}^{2}-K_{q}^{2}=\frac{\sigma^{2}(I)-\sigma_{r}^{2}-1/12}{{\left\langle I\right\rangle }^{2}},$$ (8)

where $\sigma^{2}(I)$ is the measured variance at each intensity. This PTC-based method for estimating $K_{s}^{2}$ was used in this work. Figure S2 in the Supplementary Material illustrates the advantage of this approach over using a fixed camera gain value to estimate $K_{s}^{2}$ during a human measurement.

To reduce the noise in the measured ⟨I⟩ for each window, we fit the average intensity time trace across all valid 7×7  pixel windows to the individual window’s intensity time trace and used the fitted curve to estimate the window’s ⟨I⟩.^30^^,^^36^

An intensity-squared weighted average of the $K_{f}^{2}$ values from all N windows was then calculated for each frame as follows,

$$\overset{¯}{K_{f}^{2}}=\frac{\sum_{i=1}^{N}K_{f,i}^{2}{\left\langle I\right\rangle }_{t,i}^{2}}{\sum_{i=1}^{N}{\left\langle I\right\rangle }_{t,i}^{2}},$$ (9)

where ${\left\langle I\right\rangle }_{t}$ is the temporal mean intensity of the window and subscript i denotes a quantity associated with the $i_{\mathrm{th}}$ window. Weighting the average of $K_{f}^{2}$ by the intensity reduced the error in $\overset{¯}{K_{f}^{2}}$ from camera PTC nonlinearity, especially at lower intensities.^30^^,^^33^ The BFi for SCOS was then obtained by inverting the $\overset{¯}{K_{f}^{2}}$,

$${\mathrm{BFi}}_{\mathrm{SCOS}}=1/\overset{¯}{K_{f}^{2}}.$$ (10)

The SCOS BFi, as defined above, has been shown to linearly scale with respect to the DCS BFi for exposure times much longer than the decorrelation time $\tau_{c}$.^38^^,^^62^^,^^63^ Hence, under those conditions, relative changes in SCOS and DCS BFi are expected to be the same. In our experiments, $\tau_{c}$ was measured to be tens of microseconds while the SCOS exposure time was at least 4 ms, thus satisfying the condition for linearity. Note that the SCOS exposure time was effectively equal to the laser pulse width because it was shorter than the camera exposure time (6 ms). Therefore, in this work we compared relative changes in BFi ($D_{B}$ for phantom experiments) between SCOS and DCS. From here on, we will use rBFi ($rD_{B}$ for phantom experiments) interchangeably for both SCOS and DCS to denote relative change in measured flow.

#### 2.1.5 Data acquisition strategy

For all measurements, we turned on the optical chopper and started SCOS and DCS data acquisition prior to turning on the laser and continued recording after turning off the laser. This allowed us to collect dark frames for SCOS in the same measurement and ensured that each SCOS frame acquired during laser illumination corresponded to a specific laser pulse detected by DCS. This procedure enabled precise time synchronization between SCOS and DCS measurements. We adjusted the phase of the optical chopper manually via the chopper controller box to align the laser pulses with the camera’s exposure window.

We used the open-source PsychoPy software package to set the timing of the stimuli for the human subject experiments. The PsychoPy software generated a hardware trigger signal at the start of each stimulus, which was recorded by the camera’s general-purpose I/O.

### 2.2 Phantom Experiments

**Fig. 2:**
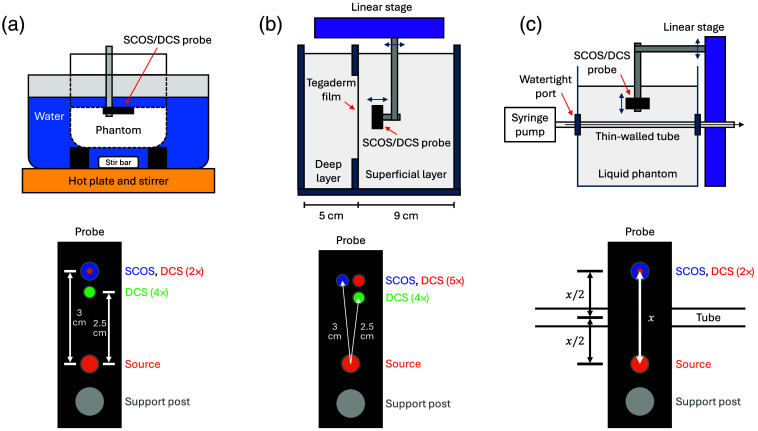
Illustrations of phantom experimental setups (top) and corresponding optical probe layouts (bottom). The number of DCS detectors averaged for each SDS is indicated in the probe drawings. (a) Temperature-ramped homogeneous phantom and corresponding probe layout. SCOS and DCS detector fibers were colocalized at 3 cm SDS. (b) Two-layer phantom and corresponding probe layout. Separate SCOS and DCS detector fibers were used at 3 cm SDS. (c) Tube-based flow phantom and corresponding probe layout. For both 2.5 and 3 cm SDSs, the tube was centered between the source and detector and oriented perpendicularly to the probe. SCOS and DCS detectors were colocalized.

The following sections describe the experimental setups for the phantom experiments, shown in Fig. 2.

#### 2.2.1 Temperature-ramped homogeneous phantom

We performed concurrent SCOS and DCS measurements on a homogeneous liquid phantom while gradually increasing its temperature from 4°C to 25°C. This temperature ramp increased the Brownian motion of the scatterers in the phantom, enabling validation of the SCOS-derived $rD_{B}$. The phantom was a mixture of water, Intralipid 20% (Fresenius Kabi), and India ink, with optical properties of $\mu_{a}=0.1\;{\mathrm{cm}}^{-1}$ and $\mu_{s}^{\prime }=9\;{\mathrm{cm}}^{-1}$ at λ=852  nm, as measured with an FD-NIRS device.^64^

As shown in Fig. 2(a), the phantom was placed in a metal container, which was then placed in a water-filled pot on a hot plate. Plastic blocks supported the phantom container, providing space for a stir bar and preventing direct heat transfer from the hot plate. The hot plate and stirrer evenly heated the surrounding water, which in turn warmed the phantom. To maintain quasi-equilibrium conditions, we limited the temperature change to no more than 1°C every 3 min. Thermistor readings at the bottom, middle, and surface of the phantom confirmed uniform heating throughout the experiment. The SCOS-DCS probe was immersed just below the phantom surface.

As shown in the probe drawing in Fig. 2(a), we used a custom Ø2.5  mm hybrid SCOS-DCS fiber bundle at 3 cm SDS,^64^ which included two single-mode DCS fibers positioned at the center of a surrounding bundle of multimode fibers for SCOS detection. We used four single-mode DCS fibers at 2.5 cm SDS.

#### 2.2.2 Two-layer phantom

The early phase of the temporal decorrelation of speckle is associated with longer pathlengths of light, which sample deeper tissue.^65^ With its high temporal sampling rate, DCS can sample the early correlation decay; including this region in the fit to the correlation function yields more depth-sensitive BFi measurements. By contrast, SCOS typically uses exposure times much longer than the speckle decorrelation time $\tau_{c}$. Although this provides the simplifying benefit of linearity between $1/K_{f}^{2}$ and $D_{B}$ as mentioned earlier, simulations have shown that it reduces the depth sensitivity of SCOS relative to DCS at a given SDS.^59^ To test this experimentally, we used a two-layer phantom with a variable superficial layer thickness to compare the depth sensitivity of SCOS and DCS.

We 3D-printed a 14  cm×14  cm×10  cm open-topped box with two compartments, as shown in Fig. 2(b), to create a two-layer liquid phantom. Each compartment was filled with a water-Intralipid-ink solution matched in optical properties ($\mu_{a}=0.15\;{\mathrm{cm}}^{-1}$ and $\mu_{s}^{\prime }=9\;{\mathrm{cm}}^{-1}$ at λ=852  nm) but differing in viscosity. The slower-diffusing layer contained 47% w/w glycerol, resulting in an approximately six-fold lower $D_{B}$ as measured by DCS—closely matching the typical cerebral-to-extracerebral blood flow ratio.^58^^,^^66^ The layers were separated by a thin, transparent medical dressing (Tegaderm), which we confirmed had no effect on $D_{B}$ measurements by testing with identical phantom compositions in both compartments. Adding glycerol increased the refractive index of the slower layer (estimated n=1.4) relative to the faster layer (n=1.33),^67^ but simulations (see details in Fig. S3(a) in the Supplementary Material) confirmed that this mismatch had negligible impact on SCOS versus DCS sensitivity comparisons. We initially tested 2% methyl cellulose as an alternative thickener due to its matched refractive index with water, but we observed that when methyl cellulose solution was added, the Intralipid tended to separate from the mixture and accumulate at the surface, causing large changes in the mixture’s scattering property (see details in Fig. S4 in the Supplementary Material).^68^ No such instability was observed when using glycerol as the thickener.

To simulate increasing the superficial tissue thickness between the probe and brain, we placed the probe in the slower-diffusing (superficial) layer and incrementally translated it away from the faster-diffusing (deep) layer. At each step, we measured $1/\overset{¯}{K_{f}^{2}}$ and $D_{B}$ using SCOS and DCS, respectively.

As shown in the probe drawing in Fig. 2(b), we used separate SCOS and DCS detector fibers at 3 cm SDS, as in the human subject experiment probe (Fig. 1). For DCS, data were averaged from five detectors at 3 cm SDS and four at 2.5 cm SDS.

#### 2.2.3 Flow phantom

Alongside depth sensitivity, noise performance is a key factor in determining the ability to resolve blood flow changes in deep tissue. To compare the combined effects of depth sensitivity and noise performance between SCOS and DCS, we conducted a tube-based flow phantom experiment. The flow phantom experiment is more representative of a focal change in blood flow within a localized brain region, as opposed to the global change modeled in the two-layer phantom experiment.

As shown in Fig. 2(c), we submerged a Ø2  mm thin-walled, transparent tube in a 15  cm×15  cm×15  cm container filled with a water-Intralipid-ink phantom ($\mu_{a}=0.1\;{\mathrm{cm}}^{-1}$ and $\mu_{s}^{\prime }=9\;{\mathrm{cm}}^{-1}$ at λ=852  nm) and pumped the same liquid phantom through the tube at a flow velocity of 2  cm/s. The tube passed through the phantom container via two watertight ports located 6 cm above the bottom. We varied the depth of the tube relative to the probe from 0.6 to 1.8 cm in 0.4-cm increments to simulate different superficial layer thicknesses. This was done for two SDSs: 2.5 and 3 cm. The tube was recentered between the source and detector after changing the SDS. As shown in the probe drawing in Fig. 2(c), we used the same custom Ø2.5  mm hybrid SCOS-DCS fiber bundle as in the temperature-ramped phantom experiment.

### 2.3 Human Study

#### 2.3.1 Participants

We recruited ten healthy subjects between the ages of 20 and 61 to undergo concurrent SCOS and DCS measurements while they performed four physiological maneuvers (breath-holding, hyperventilation, pressure modulation, and squatting) designed to elicit large, repeatable changes in blood flow. Eligibility for the study was determined based on self-reported health history, ability to provide informed consent, and vital signs assessment. The study was approved by the Mass General Brigham Institutional Review Board (#2023P002644). All participants gave written informed consent prior to the study, and all study procedures were conducted in accordance with relevant guidelines and regulations. Table S1 in the Supplementary Material reports subject demographics, self-reported skin tone, and reasons for exclusion from SCOS and/or DCS analyses.

#### 2.3.2 Breath-holding task

Subjects were seated during the measurement. After a 1-min rest period, subjects performed a 20-s end-expiration breath hold followed by 40 s of normal breathing. This sequence was repeated three more times, for a total task duration of 5 min.

#### 2.3.3 Hyperventilation task

After 1 min of rest, subjects performed audio-guided paced breathing for 1 min at a rate of 40 breaths (inhalation and exhalation) per minute. Subjects then resumed self-paced breathing for 2 min for a total task duration of 4 min.

#### 2.3.4 Pressure modulation task

After an initial 1-min rest period, a study staff member tightened a tourniquet around the subject’s head just below and adjacent to the probe for 30 s and released the tourniquet for 1 min. This sequence was repeated once more for a total task duration of 4 min.

#### 2.3.5 Squatting task

Subjects began in a standing position. After standing for 30 s, subjects held a squatting position for about 1 min and returned to a standing position for another minute. This was repeated once more for a total task duration of 5 min.

#### 2.3.6 Pulsatile waveform analysis

To compare the noise performance of SCOS and DCS *in vivo*, we quantified the noise in the recovered cardiac pulsatile waveform. We analyzed 40 cardiac cycles during a rest period for each subject. Given the high SNR of the SCOS pulsatile rBFi trace (see detalis in Fig. S5 in the Supplementary Material), we used it to identify the start and end of each cardiac cycle as well as the systolic peak.

A third-order Butterworth high-pass filter with a cutoff frequency of 0.4 Hz was applied to all SCOS and DCS traces to remove low-frequency drift.^69^ To account for heart rate variability, we truncated all cardiac pulses to match the shortest pulse duration among the 40 cycles analyzed. We did this because our data showed that heart rate variability primarily affected the length of the diastolic phase, whereas the timing of the systolic peak remained relatively constant across cycles. To enable point-by-point averaging, we linearly interpolated each pulse to ensure a consistent number of samples between the start of the pulse and the systolic peak and between the systolic peak and the end of the pulse.

## 3 Results

### 3.1 Validation Using Temperature-Ramped Homogeneous Phantom

**Fig. 3:**
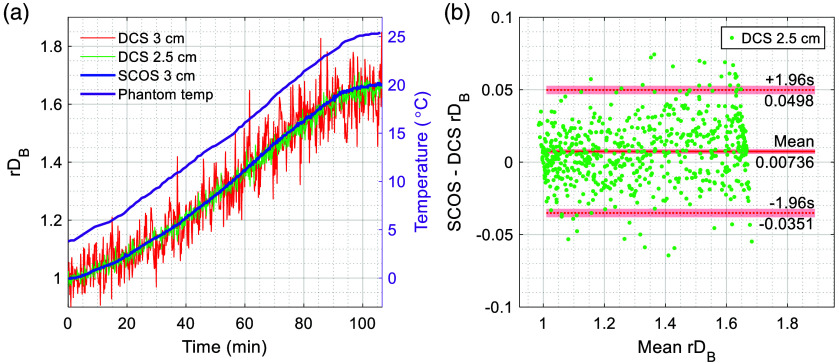
Validation of SCOS against DCS using a temperature-ramped homogeneous phantom. (a) Time traces of SCOS and DCS ${rD}_{B}$ at the indicated SDSs, along with phantom temperature. (b) Bland–Altman analysis comparing SCOS at 3 cm SDS and DCS at 2.5 cm SDS. Shaded regions represent the 95% confidence intervals for the mean difference (solid line) and the 95% limits of agreement (dotted lines).

The results of the temperature-ramped homogeneous phantom experiment [Fig. 2(a)] are shown in Fig. 3. Figure 3(a) shows the relative flow (${rD}_{B}$) measured concurrently by SCOS at 3 cm SDS and by DCS at both 2.5 and 3 cm SDS, temporally averaged to 0.1 Hz. Furthermore, the temperature of the phantom, which was recorded every minute, is depicted. The ${rD}_{B}$ tracked the increase in phantom temperature, and the alignment between SCOS and DCS $rD_{B}$ was good. Although the DCS ${rD}_{B}$ trace at 3 cm SDS was relatively noisy, as expected, it still followed the same trend as the other traces.

Figure 3(b) shows the agreement between SCOS ${rD}_{B}$ at 3 cm SDS and DCS ${rD}_{B}$ at 2.5 cm SDS through a Bland–Altman plot. Normal distribution of the differences was verified using the Kolmogorov–Smirnov test, D(640)=0.029, p=0.65. Although SCOS slightly overestimated the change in ${rD}_{B}$ relative to DCS at 2.5 cm SDS in this phantom experiment, the mean difference was within 1% and the 95% limits of agreement fell within 5%, indicating good agreement. The slight overestimation of the change in ${rD}_{B}$ by SCOS was also observed in a similar temperature-ramped phantom experiment,^43^ which was attributed to potential laser instabilities or environmental vibrations. We observed no variation in the coherence parameter β from DCS during our measurement, indicating that our laser coherence was stable. The agreement between SCOS and DCS ${rD}_{B}$ at 3 cm SDS was not included in the Bland–Altman plot due to the higher noise in the DCS measurement. Consistent with the comparison to DCS at 2.5 cm SDS, it showed a mean difference within 1%, as expected for a homogeneous phantom. The 95% limits of agreement at 3 cm SDS widened to 15% due to the increased noise in the DCS signal.

### 3.2 Comparing Depth Sensitivity Using Two-Layer Phantom

**Fig. 4:**
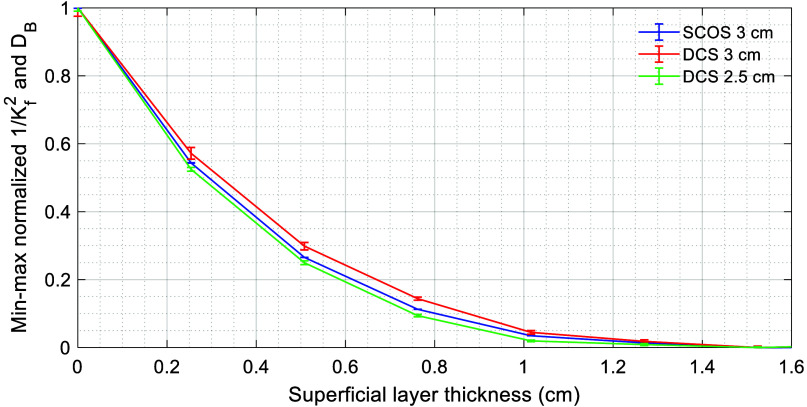
Comparison of depth sensitivity using a two-layer phantom. Shown is the min-max normalized $1/\overset{¯}{K_{f}^{2}}$ and $D_{B}$ as a function of superficial layer thickness (i.e., distance from the deep layer). Error bars represent the standard error of the mean.

To experimentally compare the depth sensitivity of SCOS and DCS, we used the two-layer phantom shown earlier in Fig. 2(b) and described in Sec. 2.2.2. We submerged the probe in the slower-diffusing (superficial) layer and varied the superficial layer thickness by incrementally translating the probe away from the interface with the faster-diffusing (deep) layer. At each step, we measured $1/\overset{¯}{K_{f}^{2}}$ and $D_{B}$ using SCOS and DCS, respectively. We min-max normalized (rescaled) the $1/\overset{¯}{K_{f}^{2}}$ and $D_{B}$ to the interval [0, 1] based on measurements at superficial layer thicknesses of 0 and 1.6 cm, where both methods were expected to be exclusively sensitive to the deep and superficial layers, respectively.^70^ Figure 4 shows the min–max normalized $1/\overset{¯}{K_{f}^{2}}$ and $D_{B}$ measured by SCOS at 3 cm SDS and by DCS at 2.5 and 3 cm SDS, respectively.

The results show that SCOS at 3 cm SDS has intermediate depth sensitivity, falling between those of DCS at 3 and 2.5 cm SDS. This agrees with prior simulations on realistic head geometries,^59^ as well as a simulation we performed on our specific two-layer phantom setup (see details in Fig. S3(b) in the Supplementary Material). In practice, DCS depth sensitivity is limited by measurement SNR, which constrains the earliest correlation delay where fitting can reliably begin.

### 3.3 Comparing Contrast-to-Noise Ratio Using Flow Phantom

**Fig. 5:**
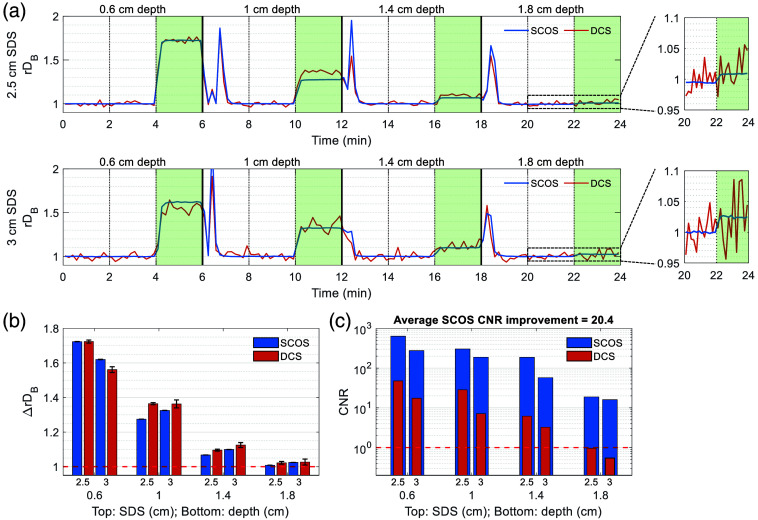
Comparison of contrast-to-noise ratio (CNR) using a tube-based flow phantom. (a) $rD_{B}$ time traces for SCOS and DCS at the different SDSs and tube depths. The subpanels show a zoomed-in view of the $rD_{B}$ time trace at 1.8 cm tube depth. Flow-on periods are highlighted with green shading. Spiking artifacts in $rD_{B}$ occurring after the flow-on periods correspond to probe adjustment and syringe refilling. (b) Flow-induced change in $rD_{B}$ as a function of SDS and depth. Error bars represent the standard error of the mean. (c) CNR as a function of SDS and depth. On average, SCOS showed a 20.4× improvement in CNR compared to the average of 2 DCS channels across all conditions tested.

To compare the contrast-to-noise ratio (CNR) of SCOS and DCS, we conducted measurements using a tube-based flow phantom with varying tube depths [Fig. 2(c)]. Figure 5(a) shows SCOS and DCS ${\mathrm{rD}}_{B}$ time traces for the two SDSs tested (2.5 and 3 cm), temporally averaged to 0.1 Hz. For each SDS, a continuous 24-min measurement was acquired while the syringe pump was turned on for 2 min at a time, and the tube depth was increased every 6 min. Spiking artifacts in the $rD_{B}$ time traces correspond to probe adjustments and syringe refilling. The impact of the lower DCS SNR was particularly evident at 3 cm SDS and 1.8 cm depth, where the flow-induced change in $rD_{B}$ was clearly captured by SCOS but obscured by noise in the DCS signal.

Figure 5(b) shows the flow-induced change in $rD_{B}$ ($\Delta rD_{B}$) measured by SCOS and DCS across all combinations of SDS and tube depth. As expected, the $\Delta rD_{B}$ decreased with increasing depth. DCS tended to recover a larger $\Delta rD_{B}$ than SCOS, consistent with its greater depth sensitivity as observed in the two-layer phantom experiment.

A partial volume effect^71^ is also evident in Fig. 5(b). As SDS increases, the photon path distribution spans a larger volume. When the tube is deep (i.e., ≥1  cm), increasing SDS increases the fraction of photons that intersect the tube, enhancing the measured flow changes. By contrast, when the tube is relatively shallow (i.e., 0.6 cm), increasing SDS reduces the fraction of the probed volume occupied by the tube, diluting the measured flow change by the unchanging background signal from the surrounding phantom. This explains the observed trend in Fig. 5(b): $\Delta rD_{B}$ decreases with increasing SDS at shallow depth (0.6 cm) but increases with SDS at deeper depth (≥1  cm). In addition, SCOS’s larger $\Delta rD_{B}$ compared with DCS at 3 cm SDS for the shallow (0.6 cm) tube depth likely reflected SCOS’s higher superficial sensitivity—a trade-off for its lower depth sensitivity.

Figure 5(c) summarizes the combined effects of depth sensitivity and noise performance using the CNR metric, defined here as,

$$\mathrm{CNR}=\Delta rD_{B}/\mathrm{CoV},$$ (11)

where CoV is the coefficient of variation during the flow-on period,

$$\mathrm{CoV}=\sigma_{rD_{B}}/\mu_{rD_{B}}.$$ (12)

Across the SDSs and depths tested, SCOS showed an average improvement in CNR of over an order of magnitude compared to DCS due to its superior noise performance. For both methods, the highest CNR was observed at the shortest SDS (2.5 cm), consistent with simulations in Ref. 59 and indicating that the gain in depth sensitivity at longer SDS was outweighed by the reduced light throughput and SNR.

### 3.4 Validation During Human Physiological Maneuvers

**Fig. 6:**
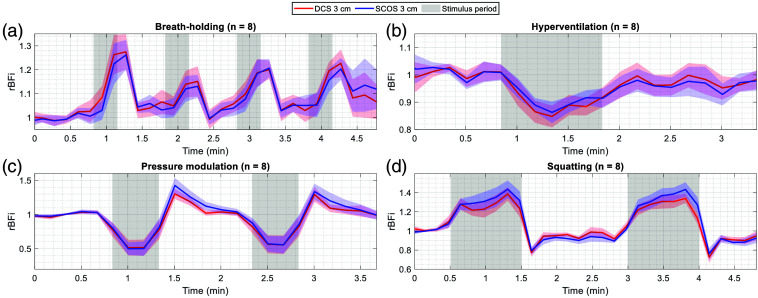
Validation of SCOS against DCS during CBF measurements on healthy volunteers performing physiological maneuvers. Shown are subject-averaged SCOS and DCS rBFi time traces at 3 cm SDS. For all maneuvers, BFi changes are relative to the baseline BFi preceding the first stimulus. The shaded area around the mean rBFi traces represents the standard error of the mean. The gray shaded regions indicate stimulus periods. (a) End-expiration breath-holding maneuver. (b) Hyperventilation maneuver, performed at 40 breaths per minute. (c) Pressure modulation maneuver using a scalp tourniquet. (d) Squatting maneuver.

To validate SCOS *in vivo*, we compared SCOS and DCS responses during controlled physiological maneuvers in healthy subjects. Figure 6 shows subject-averaged SCOS and DCS rBFi time traces at 3 cm SDS for the four maneuvers, temporally averaged to 0.1 Hz to suppress spontaneous blood flow oscillations. Two subjects were excluded due to poor DCS and/or SCOS data quality (see details in Table S1 in the Supplementary Material). For each test, baseline BFi was determined during the period preceding the first stimulus and used to calculate relative changes. DCS rBFi time traces from other SDSs (1.5, 2, and 2.5 cm) are not shown in Fig. 6 for clarity but, as an example, are shown for the hyperventilation maneuver in Fig. S6 in the Supplementary Material.

Figure 6(a) shows subject-averaged rBFi time traces during the breath-holding maneuver. Breath-holding typically leads to an increase in blood pressure^72^ and mild hypercapnia,^73^ which together cause a rise in blood flow, as observed in Fig. 6(a) and consistent with prior DCS studies.^69^^,^^74^^,^^75^

Figure 6(b) shows results for the hyperventilation maneuver. Hyperventilation is expected to cause an initial decrease in blood flow due to hypocapnia-induced vasoconstriction, followed by a compensatory increase as cerebral hypoxia triggers vasodilation to restore baseline blood flow.^76^ This biphasic response in rBFi is evident in Fig. 6(b) and aligns with observations in prior DCS studies.^69^^,^^74^^,^^75^ On the other hand, the increase in blood pressure and heart rate from hyperventilation^77^ is expected to increase scalp blood flow. Consistent with prior DCS studies,^75^^,^^78^ the DCS rBFi traces at shorter SDSs (2.5, 2, and 1.5 cm) increasingly reflected scalp hemodynamics and showed smaller reductions in measured blood flow (see details in Fig. S6 in the Supplementary Material).

Figure 6(c) shows subject-averaged rBFi time traces for the pressure modulation maneuver. Tightening of the tourniquet compresses superficial scalp vessels, reducing scalp blood flow while leaving CBF largely unaffected.^79^ Because the 3 cm SDS channels probe both cerebral and superficial compartments, a partial reduction in rBFi is expected and observed in Fig. 6(c).

Figure 6(d) shows results for the squatting maneuver. Unlike in the other tasks, subjects were standing during rest periods. Squatting increases blood pressure and CBF, which drop sharply upon returning to a standing position.^80^ This expected pattern is reflected in the rBFi traces in Fig. 6(d).

**Fig. 7:**
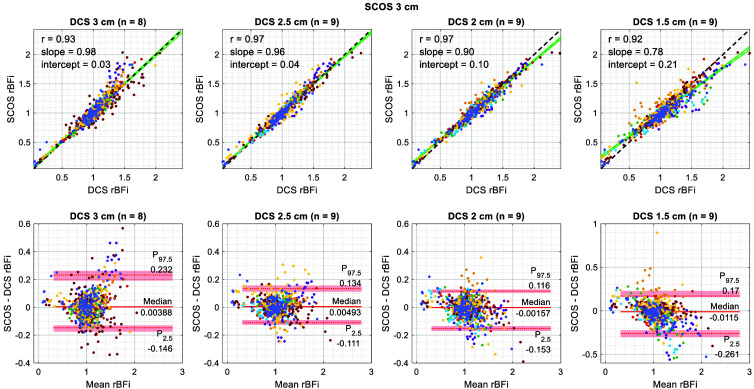
Correlation and nonparametric Bland–Altman analyses between SCOS at 3 cm SDS and DCS at every SDS tested. Data are color-coded by subject. One subject was excluded entirely due to poor SCOS and DCS data quality at all SDSs, and another subject was excluded only from the 3 cm SDS comparison due to poor DCS data quality. Each correlation plot reports the Pearson correlation coefficient, linear regression line (dashed green line), its 95% confidence interval (solid green lines), and its slope and intercept. In the Bland–Altman plots, the shaded regions indicate the 95% confidence intervals for the median difference (solid line) and the nonparametric 95% limits of agreement (dotted lines). Note that different y-axis limits were used for the comparison against DCS at 1.5 cm SDS due to the presence of an extreme outlier.

Figure 7 shows the results of correlation and Bland–Altman analyses comparing SCOS at 3 cm SDS with DCS at each SDS (1.5, 2, 2.5, and 3 cm). Data from nonexcluded subjects and all physiological maneuvers were aggregated for each comparison. One subject was excluded from the comparison at 3 cm SDS due to poor DCS data quality, but was included in the comparisons at the other SDSs. Because the differences between SCOS and DCS rBFi were not normally distributed, we performed a nonparametric Bland–Altman analysis.^81^ Specifically, we used the median of the differences to estimate the bias and the 2.5th and 97.5th percentiles of the differences to estimate the 95% limits of agreement.^82^ To estimate the 95% confidence intervals for the median and nonparametric limits of agreement, we used bootstrapping with 100,000 bootstrap samples.^82^

At 3 cm SDS, where SCOS and DCS were nearly colocalized, the Pearson correlation coefficient and linear regression slope are shown to be near unity, with a near-zero intercept, indicating good agreement. The corresponding Bland–Altman plot at 3 cm SDS shows a median difference within 1%. The correlation slope, intercept, and median difference are shown to be similar for DCS at 2.5 cm SDS, which is consistent with the finding from the two-layer phantom experiment that the depth sensitivity of SCOS at 3 cm SDS is between that of DCS at 3 and 2.5 cm SDS. The reduced noise of DCS at 2.5 cm SDS led to a higher correlation coefficient and narrower limits of agreement compared to those at 3 cm SDS.

At shorter DCS SDSs, where depth sensitivity is substantially lower than that of SCOS at 3 cm SDS, the correlation slope is shown to deviate further from unity. This trend is consistent with the expectation that the physiological maneuvers used in this study produce larger changes in scalp blood flow than in CBF. During breathing and postural tasks, CBF fluctuations are more subdued due to the stronger autoregulatory mechanisms in the brain compared to those in peripheral tissues.^83^ During the pressure modulation task, CBF is expected to remain largely unaffected while scalp blood flow is selectively suppressed. As a result, deviations in rBFi from baseline, both increases and decreases, should be more pronounced for DCS at 1.5 and 2 cm SDS, which are more sensitive to superficial tissue compared with SCOS at 3 cm SDS. This pattern is evident in both the correlation and Bland–Altman plots in Fig. 7. Additional statistics, including the 95% confidence intervals for the Pearson correlation coefficient, linear regression slope, and linear regression intercept, are provided in Table S2 in the Supplementary Material.

It should be noted that while setting the initial baseline rBFi to 1 for both SCOS and DCS might have artificially reduced the bias in our Bland–Altman analyses, this normalization was only applied to the initial baseline period. Consequently, the low bias still highlights the finding that both methods returned to the same rBFi during subsequent baseline periods, demonstrating no observable drift between the two methods (Fig. 6).

### 3.5 Comparing Noise Performance in Human Pulsatile Flow Recovery

**Fig. 8:**
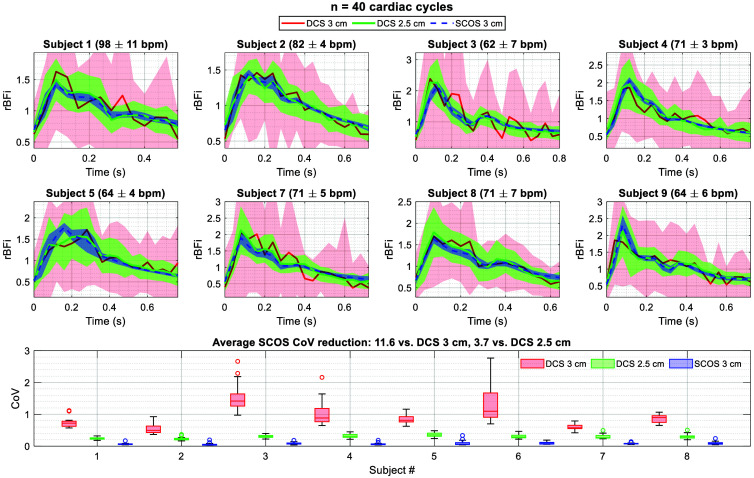
Comparison of noise in the recovered pulsatile rBFi waveform between SCOS at 3 cm SDS and DCS at both 3 and 2.5 cm SDSs. Subjects #6 and #10 were excluded due to poor DCS and/or SCOS data quality. Forty cardiac pulses recorded during a rest period were averaged for each subject. Seven channels were averaged for DCS at 3 cm SDS, and five channels were averaged for DCS at 2.5 cm SDS. Shaded regions around the mean pulsatile rBFi trace represent the standard deviation at each sampling point. The mean and standard deviation of the heart rate across the 40 cardiac cycles are reported for each subject. SCOS demonstrated an average 11.6-fold reduction in CoV compared with the seven-channel DCS at 3 cm SDS and an average 3.7-fold reduction in CoV compared with the five-channel DCS at 2.5 cm SDS.

To compare the noise performance of SCOS and DCS *in vivo*, we quantified the noise in the recovered cardiac pulsatile waveform by analyzing 40 cardiac cycles during a rest period for each subject (Fig. 8). As noted previously, the sampling rate was 25 Hz. Because we showed in the two-layer phantom experiment that the depth sensitivity of SCOS at 3 cm SDS is between those of DCS at 2.5 cm SDS and 3 cm SDS, we compared the *in vivo* noise performance of SCOS at 3 cm SDS against DCS at both 2.5 cm SDS and 3 cm SDS.

Figure 8 shows the average recovered cardiac pulse waveforms and standard deviations at each sampling point for SCOS at 3 cm SDS and DCS at both 3 cm SDS and 2.5 cm SDS. For DCS, signals from seven detectors were averaged at 3 cm SDS and five detectors were averaged at 2.5 cm SDS. The significantly improved noise performance of SCOS, over one order of magnitude higher compared to DCS at 3 cm SDS, enabled clear resolution of individual cardiac pulses (see details in Fig. S5 in the Supplementary Material). In the average SCOS waveforms, the dicrotic notch is visible in all subjects, whereas it is obscured by residual noise in the DCS 3 cm SDS averages. Despite this, the overall shape of the average pulsatile waveforms is similar between SCOS and DCS.

The box plot in Fig. 8 compares the distribution of CoVs across all sampling points in the average pulse waveform. On average, SCOS achieved an 11.6-fold reduction in CoV compared with the average of seven DCS channels at 3 cm SDS and a 3.7-fold reduction in CoV compared with the average of five DCS channels at 2.5 cm SDS. Compared with a single DCS channel, the relative reduction in SCOS CoV was 30.7 at 3 cm SDS and 8.3 at 2.5 cm SDS (assuming a square root relationship of DCS SNR with the number of detection channels).

## 4 Discussion

We report the first validation of SCOS against DCS for human CBF monitoring at a long SDS of 3 cm, across a range of physiological maneuvers. Achieving this comparison at this large separation was made possible by improvements to the SCOS system, specifically the use of a pulsed laser with higher peak power,^30^ and by averaging signals from 7 DCS detectors. We generated pulsed light by periodically blocking our wavelength-stabilized CW laser with an optical chopper, rather than by modulating the laser current as recently done in other SCOS systems.^31^^,^^84^ Although using an optical chopper led to wasted laser power and reduced adjustability of the duty cycle, it ensured stable laser coherence throughout each pulse, which was important for the accuracy of the SCOS and DCS CBF measurements in this study.

As an added note, the finite diameter of the laser beam at the optical chopper led to a 10% to 90% rise/fall time of ∼130  μs for each 4-ms (FWHM) pulse. Although the entire pulse was used for SCOS, the photon timestamps during the rising and falling transitions of the pulse were excluded from DCS analysis to prevent $g_{2}(\tau )$ distortion from nonstationary intensity.^6^^,^^85^ These photons comprised ∼7% of all photons per pulse, so removing them reduced DCS SNR by about 7% compared with the case of ideal square pulses.^59^

Proper camera calibration, which was needed to accurately correct for camera-related noise in SCOS,^36^ was also essential for demonstrating good agreement (correlation coefficient and slope >0.9) between SCOS and DCS CBF measurements in the human validation study (Fig. 7). In particular, Ref. 43 illustrated the impact of error in the measured camera gain value on SCOS BFi during an arm cuff occlusion experiment. We further illustrated in this work (Fig. S2 in the Supplementary Material) that using a fixed camera gain value may not be sufficient to accurately correct for the shot-noise-induced contrast $K_{s}$ during physiological intensity variations, due to nonlinearity in the camera’s PTC.^36^ By characterizing the camera’s nonlinear PTC and using the PTC directly to correct for $K_{s}$ [Eq. (8)], we observed that the agreement between SCOS and DCS was maintained during intensity variations. This PTC-based correction method for $K_{s}$ can improve the accuracy of SCOS measurements at lower intensities and enable the use of cameras with higher PTC nonlinearity for SCOS. In addition, we observed that our cameras, even those of the same model (Basler a2A1920-160umPRO) set to identical settings, exhibited distinct PTCs that were stable across time. Therefore, we recommend characterizing the PTC for every camera that one intends to use for SCOS.

In addition to this *in vivo* validation, we experimentally compared the depth sensitivity, noise performance, and CNR of SCOS and conventional DCS. Our two-layer phantom experiment showed that SCOS has somewhat lower depth sensitivity compared with DCS at the same SDS (Fig. 4), suggesting that DCS may retain an advantage for detecting deeper blood flow changes under certain conditions. However, the flow phantom experiment (Fig. 5) and *in vivo* pulsatile waveform comparison (Fig. 8) showed SCOS’s more-than-tenfold improvement in noise performance and CNR at the same SDS, consistent with recent Monte Carlo simulation results^59^ and demonstrating that SCOS’s advantage in SNR can outweigh its disadvantage in depth sensitivity for resolving deep flow changes. Even compared to conventional DCS at 2.5 cm SDS, SCOS at 3 cm SDS demonstrated an approximately eight-fold improvement in noise performance relative to a single DCS channel.

As DCS has been extensively validated against gold-standard CBF measurement techniques such as arterial spin labeling MRI^71^ and O^15^-positron emission tomography (PET),^86^ we used DCS as the reference modality in this study. Using DCS also allowed for concurrent, colocalized measurements with a shared laser source, enabling a more rigorous and direct comparison with SCOS. Given the strong agreement observed between SCOS and DCS in this study—and the underlying similarity between the two techniques—it appears reasonable to extend the many existing validation studies of DCS against gold-standard methods in humans to support the validity of SCOS for measuring CBF.

We used the traditional implementation of DCS in this study because it is relatively simple, robust, and still widely used in clinical research. However, in academic settings, more advanced versions of DCS have become common, particularly those using interferometric detection,^10^^–^^12^^,^^23^ longer wavelengths such as 1064 nm,^13^ highly parallelized speckle detection,^14^^–^^17^ and ToF discrimination techniques,^18^^–^^22^ all of which improve noise performance and/or depth sensitivity. Despite this, traditional DCS continues to serve as a practical benchmark. As such, the comparative validation performed in this work provides a useful reference point for assessing SCOS performance, even in relation to newer DCS implementations. We refer interested readers to Ref. 43 for a recent comparison between SCOS and a state-of-the-art interferometric diffusing wave spectroscopy (iDWS) system during microsphere phantom and human arm cuff occlusion experiments.

In a similar vein, several avenues are being actively pursued to further improve SCOS performance in terms of noise reduction and depth sensitivity. These include the use of short-pulse, high-peak-power lasers,^31^ improved optical coupling between the camera sensor and tissue,^87^ interferometric detection,^41^^,^^88^ and the implementation of ToF discrimination using either ToF detectors^47^ or interferometric detection schemes.^89^ Based on our phantom results, current SCOS depth sensitivity is limited beyond 1.5 cm, which may result in limited access to cerebral signals in some subjects.^50^ In addition, efficient methods for quantifying absolute BFi (${\mathrm{cm}}^{2}/s$) from SCOS speckle contrast would enhance the technique’s robustness for long-term CBF monitoring in dynamic clinical environments.^90^^,^^91^ Future technical and algorithmic improvements will be key to enhancing sensitivity to deeper brain regions and expanding SCOS’s potential for cerebral monitoring.

## 5 Conclusion

This work represents an important step toward establishing confidence in SCOS as a viable alternative to DCS for clinical research applications. Our results demonstrate that, with proper camera calibration and noise correction, SCOS can provide relative CBF measurements equivalent to those of DCS at long SDS (3 cm) in humans. Given its compelling combination of performance, affordability, and portability, SCOS holds strong potential for broader adoption in both research and clinical settings.

## Supplementary Material

10.1117/1.NPh.13.2.025008.s01

## Data Availability

Code, data, and 3D CAD designs used to produce the results in the main and supplementary figures are available on Harvard Dataverse (https://doi.org/10.7910/DVN/2E40FL).^92^ All other data that support the reported findings are available from the corresponding author upon reasonable request.
