# Supplementary material for: Comparative validation of speckle contrast optical spectroscopy against diffuse correlation spectroscopy for monitoring human cerebral blood flow
Source: Neurophotonics. 2026 Apr 16;13(2):025008. doi: 10.1117/1.NPh.13.2.025008 (PMC13086003; doi:10.1117/1.NPh.13.2.025008)
Supplement: Supplementary file 1 [file NPh_013_025008_SD001.pdf]

Supplemental material for comparative validation of speckle contrast optical spectroscopy against diffuse correlation spectroscopy for monitoring human cerebral blood flow

Tom Y. Cheng,[a,b,c,*] Mitchell B. Robinson,[a] Marco Renna,[a] Kuan-Cheng Wu,[a] Zachary Starkweather,[a] Olivia S. Kierul,[a] Byungchan (Kenny) Kim,[b] Alexander C. Howard,[b] David A. Boas,[b] Stefan A. Carp,[a] Xiaojun Cheng,[b] Maria Angela Franceschini[a]
[a]Athinoula A. Martinos Center for Biomedical Imaging, Massachusetts General Hospital, Department of Radiology, Boston, MA, USA
[b]Boston University, Department of Biomedical Engineering, Boston, MA, USA
[c]Lincoln Laboratory, Massachusetts Institute of Technology, Lexington, MA, USA

*Tom Y. Cheng, E-mail: tomcheng@bu.edu

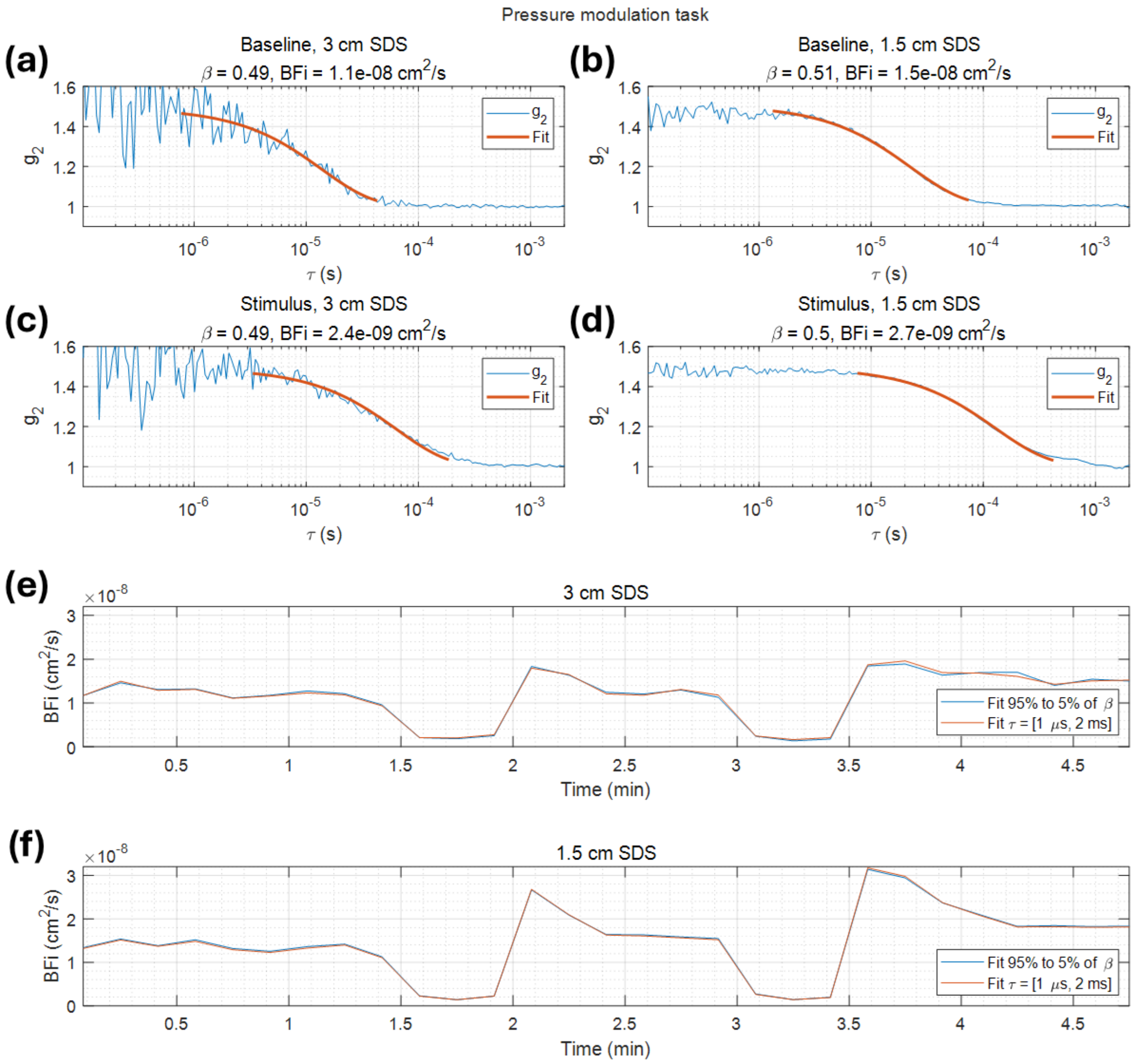

Fig. S1 Example fit of temporally averaged (0.1 Hz) $g_2(\tau)$ curves from a representative pressure modulation maneuver at 3 cm and 1.5 cm source-detector separations (SDS). In this work, we defined the fitting range as the τ interval over which $g_2(\tau) - 1$ was between 95% and 5% of the β value. Panels (a) and (b) show example $g_2(\tau)$ fits at 3 cm and 1.5 cm SDS, respectively, during baseline. Panels (c) and (d) show example $g_2(\tau)$ fits at 3 cm and 1.5 cm SDS, respectively, during pressure application. As the decay of the $g_2(\tau)$ slowed in response to a decrease in the measured blood flow during pressure application, the fitting range shifted toward later τ values. Panels (e) and (f) compare the BFi traces extracted using a dynamic fitting range (95% to 5% of β) and a fixed fitting range (1 μs ≤ τ ≤ 2 ms) at 3 cm and 1.5 cm SDS, respectively. Despite the shift in fitting range produced by the dynamic fitting method, the resulting BFi values between the two methods show negligible differences.

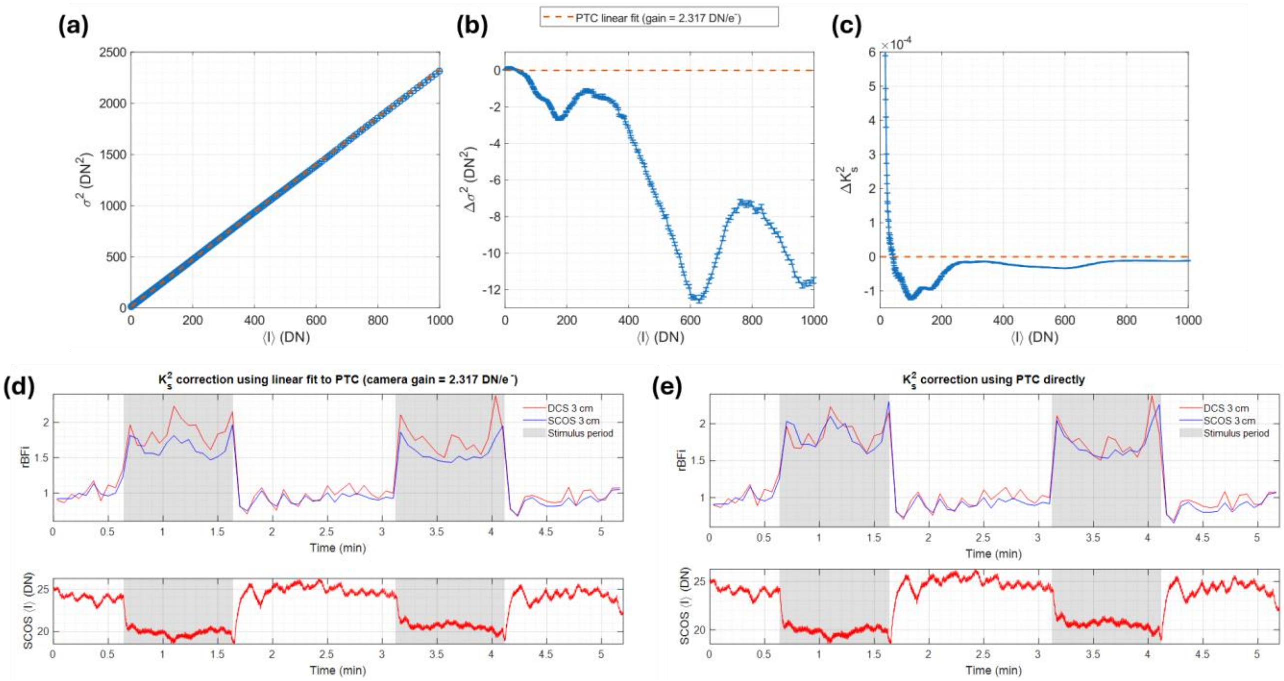

Fig. S2 Example of the effect of camera photon transfer curve (PTC) nonlinearity on a SCOS CBF measurement. Data was collected during preparation for the human subject experiments. The PTC is defined as the variance of the intensity σ^2 vs. the mean intensity $\langle I \rangle$ reading from the camera. Nonlinearity in the camera PTC introduces an intensity-dependent error in the predicted shot-noise-induced contrast, K_s, which leads to an intensity-dependent bias in the SCOS BFi. (a) Measured PTC for the Basler a2A1920-160umPRO (12-bit, 16 dB analog gain), shown for the $\langle I \rangle$ range 0–1000 DN. Error bars represent the standard error of the mean. The PTC was measured using an

automated characterization setup based largely on the setup described in Ref. 1. A camera gain value of 2.317 DN/e^- was obtained from the $1/\langle I\rangle^2$ weighted least-squares linear fit to the PTC that minimized the relative error in σ^2 (dashed line)[1]. The intercept of the fit was fixed to the dark variance (which included the read noise variance and quantization variance). (b) Residual of the variance, $\Delta\sigma^2 = \sigma^2 - \sigma_{\mathrm{fit}}^2$, where σ_{fit}^2 is the variance predicted by the linear fit to the PTC. Error bars represent the standard error of the mean. This plot illustrates the nonlinearity of the PTC in (a). (c) Error in K_s^2 due to PTC nonlinearity, $\Delta K_s^2 = \Delta\sigma^2/\langle I\rangle^2$. Error bars represent the standard error of the mean. Since $\Delta\sigma^2$ in (b) is scaled by $1/\langle I\rangle^2$ to obtain ΔK_s^2, the effect of PTC nonlinearity on ΔK_s^2 is amplified at lower $\langle I\rangle$ and attenuated at higher $\langle I\rangle$. The ΔK_s^2 increases rapidly below an intensity of ~25 DN (ΔK_s^2 above 6×10^{-4} not shown for clarity), becoming non-negligible compared to the blood flow induced K_f^2 (3×10^{-3} on average during the stimulus periods in the example measurement). A positive ΔK_s^2 means the predicted $K_s^2 = g/\langle I\rangle$ (g = 2.317 DN/e^-) is lower than the actual K_s^2 from the camera. An underestimate in K_s^2 leads to an overestimate in K_f^2, and therefore an underestimate in $\mathrm{BFi}_{\mathrm{SCOS}} = 1/K_f^2$. Panels (d) and (e) show SCOS and DCS rBFi time traces, as well as SCOS mean intensity, from the same human squatting maneuver analyzed using two different approaches to correct for K_s^2: (d) calculating $K_s^2 = g/\langle I\rangle$ for each 7×7 window using a fixed camera gain value of g = 2.317 DN/e^- obtained from the linear fit to the PTC, and (e) calculating $K_s^2 = \frac{\sigma^2(I)-\sigma_r^2-1/12}{\langle I\rangle^2}$ for each 7×7 window using $\sigma^2(I)$ from the PTC in (a), where σ_r^2 is the average read noise variance of the window. The same pixels were analyzed for both approaches, as evidenced by the identical $\langle I\rangle$ traces. The intensity change during the squatting period caused SCOS to deviate from DCS in (d), while agreement was maintained in (e). Note that, to better illustrate the effect of PTC nonlinearity in this example, a simple average of K_f^2 across 7x7 windows in the image was taken for both approaches rather than an intensity-squared weighted average of K_f^2.

Table S1 Subject demographics and data quality notes. Each subject's gender, age, race, and self-reported skin tone (Fitzpatrick scale) are listed. The final column documents any data quality issues that led to exclusion of SCOS or DCS data from analysis.

Subject #	Gender	Age	Race	Skin tone (self-reported)	Reasons for exclusion
1	Female	23	White	Type I (light)	N/A
2	Male	38	Black	Type VI (dark)	N/A
3	Male	24	White	Type I (light)	N/A
4	Male	61	White	Type II (light)	N/A
5	Female	43	Asian	Type III (medium)	N/A
6	Male	29	White	Type II (light)	Excluded DCS 3 cm SDS data. Poor data quality caused by low photon count rate (<2 kcps)
7	Male	33	Asian	Type IV (medium)	N/A
8	Male	33	White	Type I (light)	N/A
9	Female	20	White	Type I (light)	N/A
10	Female	24	Asian	Type II (light)	Excluded all SCOS and DCS data. Persistent, high-amplitude artifacts caused by accidental probe placement over hair

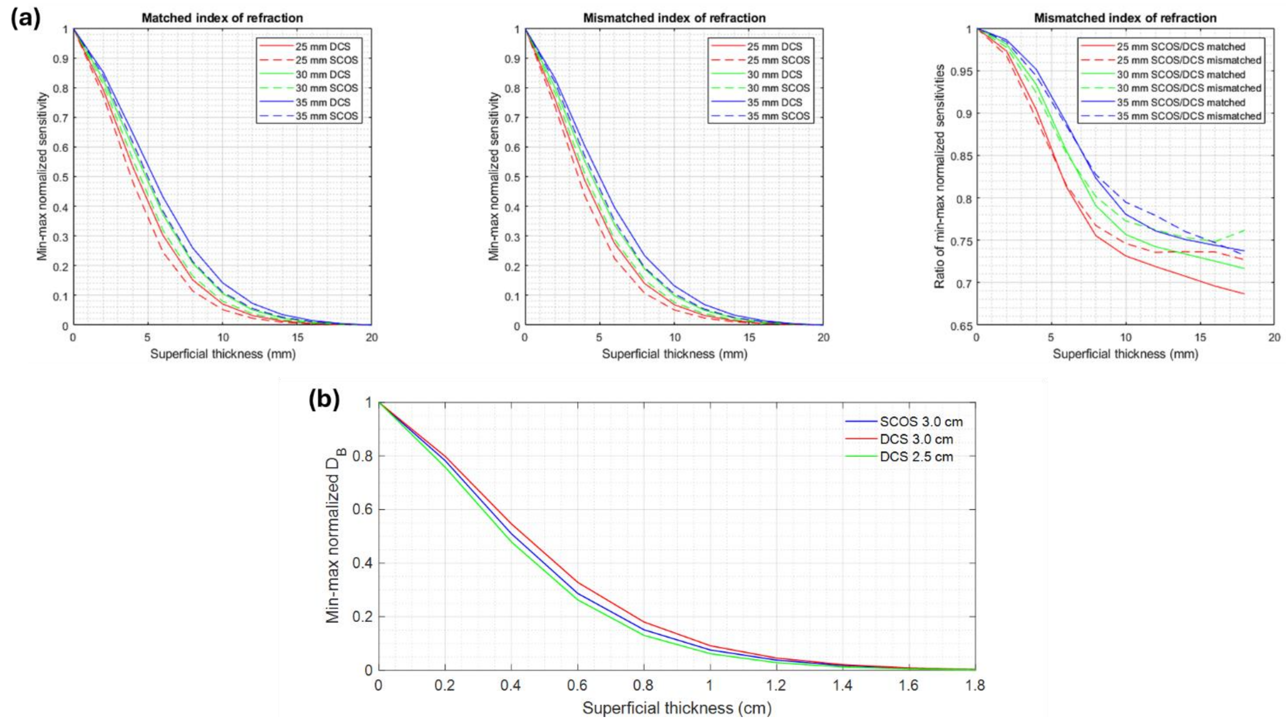

Fig. S3 Monte Carlo simulation of the two-layer phantom using parameters matched to the experimental phantom setup. (a) Simulated effect of refractive index mismatch between superficial and deep layers. While the mismatch reduces the absolute sensitivity to the deep layer, it has little impact on the relative sensitivity of SCOS vs. DCS. (b) Simulated min-max normalized D_B of SCOS at 3 cm SDS and DCS at 3 cm and 2.5 cm SDS. The relative sensitivity of SCOS vs. DCS in simulation is consistent with the experimental results shown in Fig. 4.

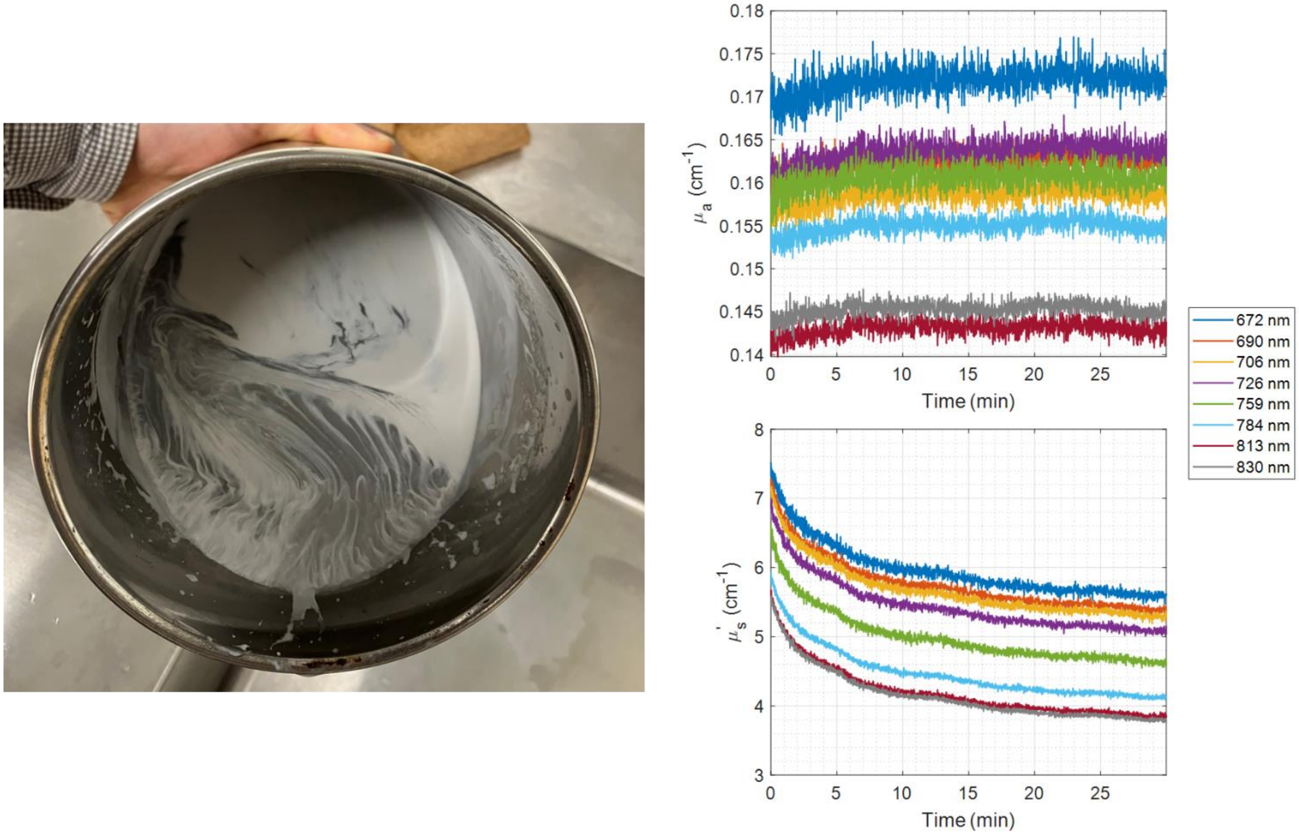

Fig. S4 Layering effect in water-methylcellulose-Intralipid-ink phantom. The left image shows separation of the Intralipid from the mixture and the formation of a concentrated lipid layer at the surface, after the phantom was left undisturbed. The layering effect primarily caused instability in the reduced scattering coefficient (μ_s'). Shown on the right are time traces of the absorption coefficient (μ_a) and reduced scattering coefficient at various wavelengths following phantom stirring. Optical properties were measured with a frequency-domain near-infrared spectroscopy system (MetaOx, ISS)[2] using a multi-distance probe immersed just below the surface. While μ_a remained relatively stable over time, μ_s' decreased substantially due to sedimentation or separation of scattering components, by approximately 30% over 30 minutes. Although the rate of decline slowed over time, it did not plateau, indicating ongoing instability in the scattering properties of the phantom.

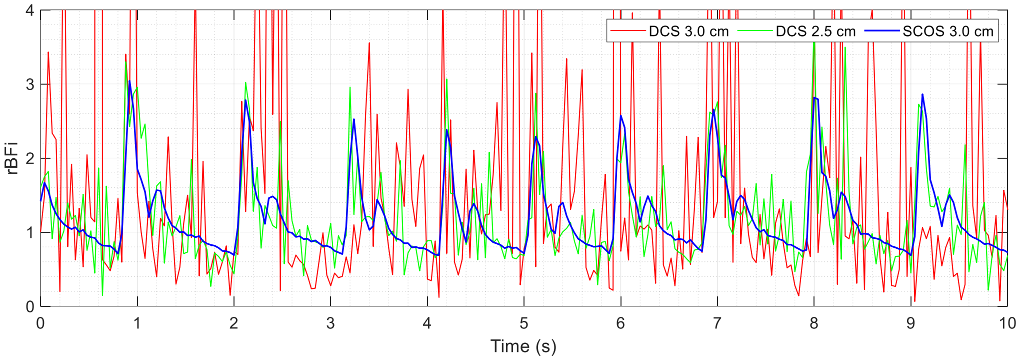

Fig. S5 Example pulsatile rBFi waveform from a single subject. Sampling rate was 25 Hz. SCOS and DCS traces were measured at 3 cm SDS, with SCOS showing clearly resolved cardiac pulsations. For DCS, signals were averaged across seven detectors at 3 cm SDS and five detectors at 2.5 cm SDS. This example illustrates the higher signal-to-noise ratio of SCOS compared to DCS for resolving pulsatile flow.

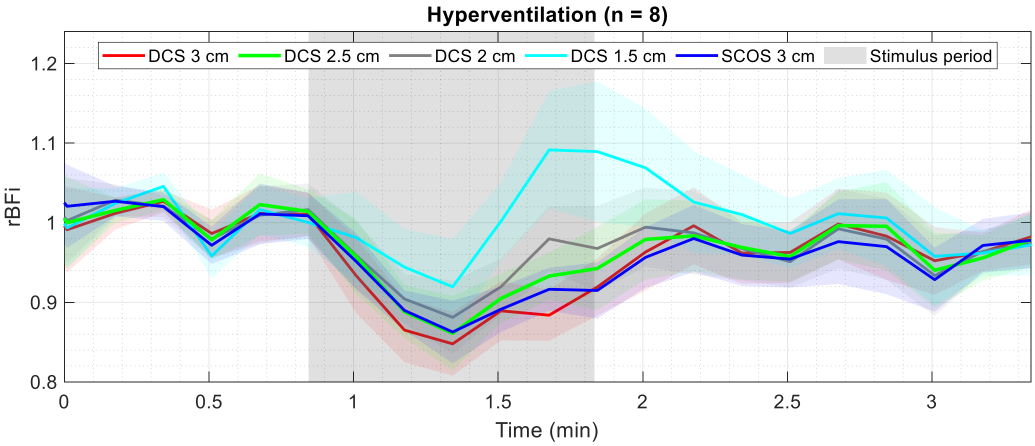

Fig. S6 Subject-averaged SCOS and DCS rBFi time traces for the hyperventilation maneuver (performed at 40 breaths per minute), temporally averaged to 0.1 Hz to suppress spontaneous blood flow oscillations. Two subjects were excluded due to poor data quality. For DCS, signals were averaged across seven detectors at 3 cm SDS, five detectors at 2.5 cm SDS, and two detectors at 2 cm SDS. A single detector was used at 1.5 cm SDS. The shaded area around the mean rBFi traces represent the standard error of the mean, and the gray shaded region indicates the stimulus period. The DCS rBFi at shorter SDSs increasingly reflected scalp hemodynamics and showed smaller reductions in measured blood flow. The mean rBFi of SCOS at 3 cm SDS was between those of DCS at 2.5 cm SDS

and 3 cm SDS during the hyperventilation maneuver, consistent with the depth sensitivity results from the two-layer phantom experiment (Fig. 4 and Fig. S3).

Table S2 Correlation and linear regression statistics for the comparison between SCOS and DCS rBFi during human physiological maneuvers. The tabulated values correspond to the correlation results shown in Fig. 7. SDS = source-detector separation, CI = confidence interval.

**DCS SDS (cm)**	**Regression slope**		**Regression intercept**		**Pearson correlation coefficient**		
	Estimate	95% CI	Estimate	95% CI	Estimate	P-value	95% CI
3.0	0.98	[0.96, 1.01]	0.03	[0.00, 0.05]	0.93	<.001	[0.92, 0.94]
2.5	0.96	[0.95, 0.98]	0.04	[0.03, 0.06]	0.97	<.001	[0.97, 0.98]
2.0	0.90	[0.88, 0.91]	0.10	[0.08, 0.11]	0.97	<.001	[0.96, 0.97]
1.5	0.78	[0.76, 0.80]	0.21	[0.19, 0.23]	0.92	<.001	[0.92, 0.93]

References

1. T. Y. Cheng et al., "Choosing a camera and optimizing system parameters for speckle contrast optical spectroscopy," Sci. Rep. **14**(1), 11915, Nature Publishing Group (2024) [doi:10.1038/s41598-024-62106-y].
2. S. A. Carp et al., "Combined multi-distance frequency domain and diffuse correlation spectroscopy system with simultaneous data acquisition and real-time analysis," Biomed. Opt. Express **8**(9), 3993–4006, Optica Publishing Group (2017) [doi:10.1364/BOE.8.003993].

DISTRIBUTION STATEMENT A. Approved for public release. Distribution is unlimited. This material is based upon work supported under Air Force Contract No. FA8702-15-D-0001 or FA8702-25-D-B002. Any opinions, findings, conclusions or recommendations expressed in this material are those of the author(s) and do not necessarily reflect the views of the U.S. Air Force.
